# The rise in vancomycin-resistant *Enterococcus faecium* in Germany: data from the German Antimicrobial Resistance Surveillance (ARS)

**DOI:** 10.1186/s13756-019-0594-3

**Published:** 2019-08-28

**Authors:** Robby Markwart, Niklas Willrich, Sebastian Haller, Ines Noll, Uwe Koppe, Guido Werner, Tim Eckmanns, Annicka Reuss

**Affiliations:** 1 Robert Koch Institute, Department 3: Infectious Disease Epidemiology, Unit 37: Healthcare-associated Infections, Surveillance of Antibiotic Resistance and Consumption, Robert Koch Institute, Nordufer 20, 13353 Berlin, Germany; 2 Robert Koch Institute, Department 3: Infectious Disease Epidemiology, Unit 34: HIV/AIDS, STI and Blood-borne Infections, Robert Koch Institute, Nordufer 20, 13353 Berlin, Germany; 3 Robert Koch Institute, Department 1: Infectious Diseases, Unit 13: Nosocomial Pathogens and Antibiotic Resistances, Robert Koch Institute, Nordufer 20, 13353 Berlin, Germany

**Keywords:** *Enterococcus faecium*, Vancomycin resistance, Antimicrobial resistance, Surveillance, Epidemiology, ARS

## Abstract

**Background:**

Due to limited therapeutic options, vancomycin-resistant *Enterococcus faecium* (VREF) is of great clinical significance. Recently, rising proportions of vancomycin resistance in enterococcal infections have been reported worldwide. This study aims to describe current epidemiological trends of VREF in German hospitals and to identify factors that are associated with an increased likelihood of vancomycin resistance in clinical *E. faecium* isolates.

**Methods:**

2012 to 2017 data from routine vancomycin susceptibility testing of 35,906 clinical *E. faecium* isolates from 148 hospitals were analysed using data from the German *Antimicrobial Resistance Surveillance* System. Descriptive statistical analyses and uni- and multivariable regression analyses were performed to investigate the impact of variables, such as year of sampling, age and region, on vancomycin resistance in clinical *E. faecium* isolates.

**Results:**

From 2014 onwards the proportions of clinical *E. faecium* isolates exhibiting resistance to vancomycin increased from 11.2% (95% confidence interval [CI] 9.4–13.3%) to 26.1% (95% CI 23.1–29.4%) in 2017. The rise of VREF proportions is primarily observed in the southern regions of Germany, whereas northern regions do not show a major increase. In the Southwest and Southeast, VREF proportions increased from 10.8% (95% CI 6.9–16.5%) and 3.8% (95% CI 3.0–11.5%) in 2014 to 36.7% (95% CI 32.9–40.8%) and 36.8% (95% CI 29.2–44.7%) in 2017, respectively. VREF proportions were considerably higher in isolates from patients aged 40–59 years compared to younger patients. Further regression analyses show that in relation to secondary care hospitals, *E. faecium* samples collected in specialist care hospitals and prevention and rehabilitation care centres are more likely to be vancomycin-resistant (odds ratios: 2.4 [95% CI 1.2–4.6] and 2.4 [95% CI 1.9–3.0], respectively). No differences in VREF proportions were found between female and male patients as well as between different clinical specimens.

**Conclusion:**

The proportion of VREF is increasing in German hospitals, particularly in southern regions in Germany. Increased efforts in infection control and antibiotic stewardship activities accounting for local resistance patterns are necessary to combat the spread of VREF in Germany.

**Electronic supplementary material:**

The online version of this article (10.1186/s13756-019-0594-3) contains supplementary material, which is available to authorized users.

## Background

*Enterococcus faecium* is a Gram-positive, facultative anaerobic, catalase-negative bacterium that commonly inhabits the intestinal tracts of healthy humans [1]. In addition to its role as a commensal in humans, *E. faecium* has been described as an emerging pathogen that causes a significant number of nosocomial infections, including infections of the bloodstream, urinary tract, skin and endocardium [2]. Data from the United States [3] and Germany [4] show that *E. faecium* is among the most frequent causes of healthcare-associated infections with considerable potential for healthcare-acquired outbreaks. Evidence indicates that *E. faecium* strains that cause nosocomial infections are different from strains that colonize healthy humans highlighting the role of healthcare centres in the spread of *E. faecium* infections [5–7].

The clinical relevance of *E. faecium* is directly linked to its intrinsically low susceptibility to a broad spectrum of antimicrobial agents, including low-dose penicillin and ampicillin, aminoglycosides, sulphonamides and cephalosporines [8, 9]. After its first detection in the late 1980’s, vancomycin resistance in *Enterococci* (VRE), including *E. faecium*, started to emerge in hospitals in the United States eventually spreading to Europe and worldwide limiting therapeutic options against enterococcal infections [10]. Due to its clinical significance, the *World Health Organization* (WHO) assigned vancomycin-resistant *E. faecium* (VREF) as a high priority pathogen on its global priority list of antibiotic-resistant bacteria [11].

According to 2017 data from the European Antimicrobial Resistance Surveillance Network (EARS-Network), the mean proportion of vancomycin-resistant *E. faecium* in blood and cerebrospinal fluid isolates is 14.9% (95% CI 14–16) in participating European countries and 16.5% (95% CI 15–18) in Germany [12]. Findings from the German national nosocomial infection surveillance system (*Krankenhaus-Infektions-Surveillance-System*, acronymized as “KISS”) show continuously increasing rates of vancomycin-resistant *Enterococci* from nosocomial bloodstream and urinary tract infections acquired in intensive care units (ICU) between 2007 and 2016 [13].

Despite the data available for Germany, a comprehensive picture of the epidemiological situation of vancomycin-resistant *E. faecium* in German hospitals is lacking. In particular, it is not known whether distinct patient characteristics (e.g. gender, age, site of infection) or other factors (e.g. hospital care type) are associated with an increased risk of VREF. Therefore, this study aims to analyse trends and risk factors of vancomycin resistance of *Enterococcus faecium* in Germany using data from the German national *Antimicrobial Resistance Surveillance* (ARS) System. Furthermore, the study analyses trends in the number of infections or colonisations with VREF diagnosed in German hospitals using publicly available data from the German hospital payment system based on fee-for-case on diagnosis related groups.

## Methods

### Study design and the German antimicrobial resistance surveillance database

In order to investigate the epidemiology of vancomycin-resistant *E. faecium*, a retrospective observational study was conducted analysing data from the *Antimicrobial Resistance Surveillance* (ARS) database from 2012 to 2017. ARS is the national surveillance system for antimicrobial resistance in Germany established by the Robert Koch-Institute in 2008 [14]. Voluntarily participating microbiology laboratories submit results from routine pathogen identifications and antimicrobial susceptibility testing. In addition to microbiological results, participating laboratories provide various pseudonymised information including clinical specimen material (e. g. blood, urine and swabs), patient data (age, gender), hospital type (e. g. secondary or tertiary care hospitals) and geographical location of patient care [15, 16]. As of 2017, more than 50 laboratories contribute to the ARS database, which includes data from more than 600 out of a total of 1924 hospitals in Germany. Since ARS participation is based on laboratories rather than active participation of hospitals, a major selection bias towards certain hospitals (e.g. only those with implemented antibiotic stewardship programs) can be excluded. All participating laboratories possess accreditation for performing microbiological analyses. Data transmitted to the ARS database are routinely validated and checked for plausibility, completeness and consistency. ARS data are used to generate reference resistance data and feedback reports to support hospitals in their antibiotic stewardship programs. ARS resistance data of common pathogens are also available to the public (https://ars.rki.de/).

### Selection of *E. faecium* isolates

The participation of individual laboratories in ARS can change over time which can potentially alter the set of hospitals that provide clinical samples to ARS. In order to avoid systematic changes in the case mix, only *E. faecium* isolates from hospitals with continuous yearly participation in ARS between 2012 and 2017 were included for the main analyses. To avoid biases through inclusion of multiple *E. faecium* isolates from one patient during one disease episode, only the patient’s first isolate for each quarter of the year was included. However, since vancomycin-resistant enterococci are known to persist in the human gut for several months [17], it cannot be fully excluded that a specific VREF strain has been counted repeatedly from the same patient. Furthermore, isolates were excluded if they were likely derived for screening purposes (labelled as screening, anal swabs and stool samples). *E. faecium* isolates without vancomycin susceptibility testing were excluded.

### Outcomes and co-variables

The primary outcome is the proportion of vancomycin-resistant *E. faecium* isolates among all *E. faecium* isolates expressed as percentages (%). An *E. faecium* isolate was defined vancomycin resistant if it was tested resistant against vancomycin in antimicrobial susceptibility testing according to the applied standard, i. e. standards by *European Committee on Antimicrobial Susceptibility Testing* (EUCAST) or *Clinical and Laboratory Standards Institute* (CLSI).

Clinical specimens were grouped by sample site into urine (urine samples), blood (blood cultures), swabs (swabs from eye, nose, throat, ear, tongue, urogenital sites as well as intraoperative swabs and other/unspecified swabs), wound (swabs from wounds and abscesses) and other specimens (e. g. punctures, respiratory materials, unspecified). Patient age was grouped into age categories (0–19, 20–39, 40–59, 60–79 and, ≥80 years). Patient gender was classified into female and male. The geographical origin of the isolates was grouped into five major regions based on the distribution of hospitals: Northeast (federal states of Mecklenburg-West Pomerania, Brandenburg, Berlin, Saxony-Anhalt), Northwest (federal states of Lower Saxony, Bremen, Hamburg, Schleswig-Holstein), West (North Rhine-Westphalia), Southwest (Hesse, Rhineland-Palatinate, Saarland, Baden-Wuerttemberg) and Southeast (Bavaria, Saxony, Thuringia). Hospital care type was categorised into secondary care, tertiary care, specialist care, and prevention and rehabilitation care. All variables were considered as categorical variables for statistical analyses.

### Statistical analyses

All statistical analyses were performed using R version 3.5.1 [18]. Estimates of vancomycin resistance proportions are expressed as percentages with 95% confidence intervals (95% CI) accounting for clustering on hospital level using routines in the *survey* package (version 3.35). Proportions of vancomycin-resistant *E. faecium* isolates between female and male were compared using the Pearson χ^2^ test with the Rao-Scott second-order correction [19] for different age groups. The resulting *p*-values were adjusted for multiple testing using a Bonferroni correction. Risk factors for vancomycin resistance were analysed using univariable and multivariable logistic regression models accounting for clustering at hospital level as implemented in the survey package. For univariable analyses the following predictors for vancomycin resistance were considered: year of sampling, gender, age group, specimen (sample site), region and hospital care type. The multivariable analysis model included all variables from the univariable analyses. For the multivariable analysis assessing the interaction between region and year of sampling, the year was treated as a continuous predictor and the interaction between region and year was included. The same variables as in the model without interaction were otherwise included.

### Sensitivity analyses

It is important to note that some laboratories do not routinely differentiate *Enterococcus* isolates into species level. Systematic bias in VREF proportions therefore cannot be excluded, such as those introduced by species differentiation only in selected *Enterococcus* samples. To address this issue, time trend analyses of VREF proportions were analysed for *E. faecium* isolates identified in laboratories that consistently differentiate more than 95% of all *Enterococcus* isolates into species level (*n* = 8492). In addition, sensitivity analyses were performed that comprised *E. faecium* isolates (*n* = 89,450) from all hospitals including hospitals that did not continuously participated in ARS between 2012 and 2017.

### Data from the hospital payment system based on fee-for-case on diagnosis related groups

In order to estimate the number of diagnosed infections or colonisations with VREF between 2013 and 2017, publicly available data from the hospital payment system based on fee-for-case on diagnosis related groups (DRG) were analysed. German hospitals receive a fee-for-case on DRG based on diagnoses according to the *International Statistical Classification of Diseases and Related Health Problems Version 10 - German Modification* (ICD-10-GM). According to §21 Hospital Reimbursement Act (Krankenhausentgeltgesetz) aggregated data must be made publicly available for scientific use by the *Insitute for Reimbursement in the Hospital* (*Institut für das Entgeltsystem im Krankenhaus, InEK)* [20]. The dataset contains diagnosis data from approximately 1500 out of 1924 German hospitals. The diagnosis code U80.30 (*E. faecium* with resistance to glycopeptide antibiotics, available since 2013) was used to identify cases of *E. faecium* with resistance to glycopeptide antibiotics. Importantly, a diagnosis code for *E. faecium* with resistance to glycopeptide antibiotics has been implemented since the beginning of the DRG system in Germany in 2004 (U.80.3!: *E. faecium* with resistance to glycopeptide antibiotics, oxazolidinone, streptogramine, or high-level-aminoglycoside-resistance). Therefore, the diagnosis of glycopeptide-resistant *E. faecium* is well established in German hospitals and a reporting bias through introduction of the fee-for-case on DRG can be excluded.

## Results

### Baseline characteristics

In total 35,906 *E. faecium* isolates from 33,643 patients and 148 continuously participating hospitals were included in the study. The baseline characteristics are outlined in Table 1. Samples predominantly originated from elderly patients (median: 74 years), although isolates from younger age categories were also available. With a female / male ratio of 1.16 patients’ gender was nearly equally distributed in the sample set. The majority of hospitals and isolates originated from Western and Southwestern regions in Germany, regions where the most populated federal states are located, including North Rhine Westphalia (~ 18 m inhabitants) and Baden-Wuerttemberg (~ 11 m). The largest number of *E. faecium* isolates was provided by secondary care hospitals (*n* = 31,182) followed by tertiary care hospitals (*n* = 3.283), and specialist care hospitals (1109). The most common clinical sources of *E. faecium* were urine samples (*n* = 16,261), swabs (*n* = 5687) and wound material (*n* = 5550). It is worth mentioning that the ratio of the total numbers of clinical *E. faecium* and *E. faecalis* isolates recorded in ARS did not change between 2012 and 2017 (Additional file 1: Table S1). Compared to other regions in Germany, in the West and Southwest slightly higher proportions of *E. faecium* were observed.

**Table 1:** Baseline characteristics of clinical *E. faecium* isolates

*N*_*total*_	35,906	
*Year of sampling*		
2012 (n, %)	4139	11.53
2013 (n, %)	5452	15.18
2014 (n, %)	6326	17.62
2015 (n, %)	6932	19.31
2016 (n, %)	6806	18.96
2017 (n, %)	6251	17.41
*Gender of patient*		
Female (n, %)	17,892	49.83
Male (n, %)	15,370	42.81
NA (n, %)	2644	7.36
Sex ratio (f/m)	1.16	
*Age of patient*		
0–19 yrs. (n, %)	527	1.47
20–39 yrs. (n, %)	1183	3.29
40–59 yrs. (n, %)	5547	15.45
60–79 yrs. (n, %)	17,785	49.53
80+ yrs. (n, %)	10,864	30.26
Age (median, IQR)	74.0	63.0–81.0
*Specimen (sampling site)*		
Blood (n, %)	3011	8.39
Urine (n, %)	16,261	45.29
Swab (n, %)	5687	15.84
Wound (n, %)	5550	15.46
Other (n, %)	5280	14.71
NA (n, %)	117	0.33
*Region*		
Southwest (n, %)	11,868	33.05
Southeast (n, %)	395	1.10
West (n, %)	19,508	54.33
Northwest (n, %)	2400	6.68
Northeast (n, %)	1594	4.44
NA (n, %)	141	0.39
*Hospital care type*		
Secondary care (n, %)	31,182	86.84
Tertiary care (n, %)	3283	9.14
Specialist care	1109	3.09
Prevention and rehabilitation care (n, %)	158	0.44
Other (n, %)	33	0.09
NA (n, %)	141	0.39
*Hospitals (n)*	148	
*Patients (n)*	33,643	

### Temporal trend and regional analyses

**Fig. 1:**
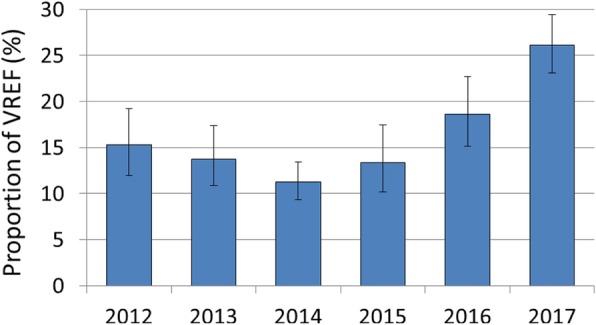
Time trend of vancomycin-resistant *E. faecium.* Time trend of vancomycin-resistant *E. faecium* as a proportion (%) of all *E. faecium* isolates with corresponding 95% confidence intervals

The proportion of *E. faecium* isolates with resistance against vancomycin decreased from 15.2% (95% CI 12.0–19.2%) in 2012 to 11.2% (95% CI 9.4–13.4%) in 2014 (Fig. 1). However, from 2014 onwards, the percentage of vancomycin-resistant clinical *E. faecium* isolates continuously increased reaching 26.1% (95% CI 23.1–29.4%) in 2017, more than twice that observed 2014. This finding is supported by univariable and multivariable analyses, which show that isolates collected after 2014 were increasingly more likely to be tested resistant against vancomycin than isolates in 2014 (Table 2). A similar rise of VREF proportions between 2014 and 2017 was found in sensitivity analyses including *E. faecium* isolates that (i) were identified in laboratories that consistently differentiate more than 95% of all *Enterococcus* isolates into species level (Additional file 2: Figure S1A) or (ii) were provided by all hospitals also including hospitals that did not continuously participated in ARS between 2012 and 2017 (Additional file 2: Figure S1B). Since bloodstream infections are of particular clinical interest, it is noteworthy that in the included hospitals the number of VREF blood isolates increased from 57 to 120 between 2014 and 2017 accompanied by a marked rise in VREF proportions from 11.0% (95% CI 7.2–16.6) in 2014 to 21.1% (95% CI 17.2–25.7%) in 2017.

**Table 2:** Uni- and multivariable analyses of factors associated with vancomycin resistance in clinical *E. faecium* isolates

	univariable analysis			multivariable analysis		
	OR	(95% CI)	*p*-value	OR	(95% CI)	*p*-value
*Year of sampling*						
2012	1.42	(1.12–1.80)	0.004	1.58	(1.16–2.14)	0.004
2013	1.26	(1.01–1.58)	0.044	1.27	(0.99–1.63)	0.061
2014	1	–	–	1	–	–
2015	1.22	(0.98–1.51)	0.072	1.24	(0.99–1.55)	0.060
2016	1.81	(1.46–2.23)	< 0.001	1.80	(1.45–2.25)	< 0.001
2017	2.79	(2.26–3.46)	< 0.001	2.74	(2.22–3.39)	< 0.001
*Region*						
Southwest	1	–	–	1	–	–
Southeast	0.71	(0.53–0.95)	0.022	0.74	(0.54–1.01)	0.057
West	0.68	(0.46–1.00)	0.051	0.82	(0.55–1.20)	0.303
Northwest	0.46	(0.35–0.61)	< 0.001	0.51	(0.36–0.72)	< 0.001
Northeast	0.43	(0.27–0.67)	< 0.001	0.47	(0.31–0.71)	0.001
*Gender*						
Female	1	–	–	1	–	–
Male	0.97	(0.90–1.06)	0.533	1.03	(0.94–1.11)	0.554
*Age*						
0–19 years	0.38	(0.26–0.58)	< 0.001	0.33	(0.23–0.48)	< 0.001
20–39 years	0.85	(0.71–1.01)	0.071	0.84	(0.71–0.99)	0.043
40–59 years	1	–	–	1	–	–
60–79 years	0.96	(0.82–1.12)	0.626	0.94	(0.83–1.06)	0.324
80+ years	0.86	(0.67–1.10)	0.234	0.83	(0.69–1.01)	0.062
*Specimen (sampling site)*						
Blood	1	–	–	1	–	–
Urine	1.18	(0.92–1.52)	0.20276	1.22	(0.94–1.48)	0.134
Swab	1.09	(0.78–1.51)	0.62974	0.93	(0.74–1.17)	0.547
Wound	1.12	(0.93–1.36)	0.22761	1.08	(0.90–1.29)	0.426
Other	1.04	(0.88–1.23)	0.65102	1.01	(0.85–1.19)	0.952
*Hospital care type*						
Secondary care	1	–	–	1	–	–
Tertiary care	1.65	(1.01–2.69)	0.047	1.33	(0.87–2.04)	0.191
Specialist care	2.53	(1.15–5.56)	0.023	2.37	(1.22–4.60)	0.012
Prevention and rehabilitation care	3.23	(2.42–4.33)	< 0.001	2.39	(1.91–2.98)	< 0.001

**Fig. 2:**
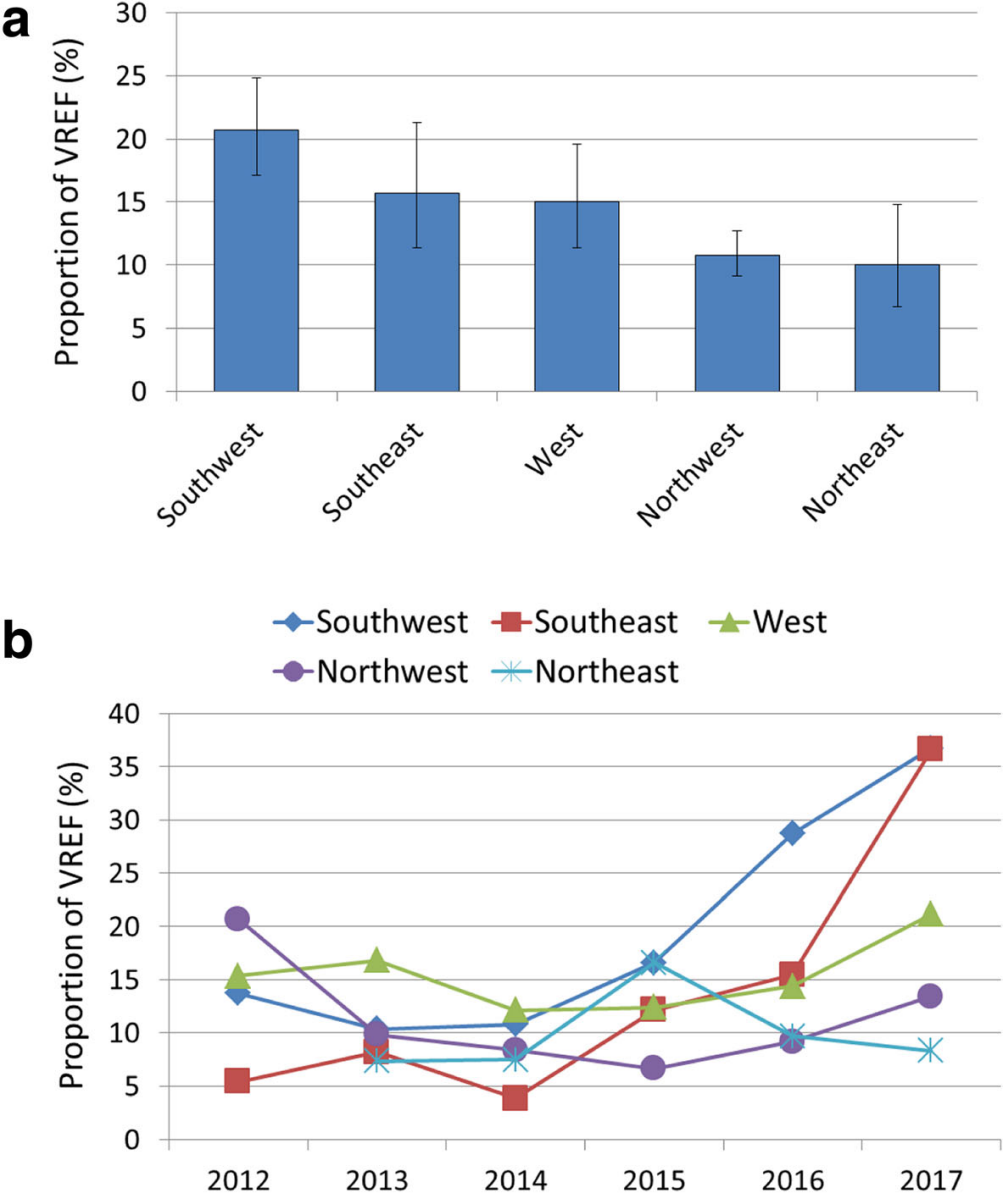
Vancomycin-resistant *E. faecium* stratified into geographical region. Vancomycin-resistant *E. faecium* as a proportion (%) of all *E. faecium* isolates with corresponding 95% confidence intervals by German region (2012–2017 data) (**a**) and time trend by German region (**b**)

Regional analyses of *E. faecium* isolates collected between 2012 and 2017 reveal that Germany shows a strong north-south disparity in VREF proportions. VREF proportions are noticeably lower in the Northwest (10.8% [95% CI 6.7–14.8]) and Northeast (10.0% [95% CI 6.7–14.8]) compared to the Southwest where 20.7% (95% CI 17.1–24.9) of all isolates were tested resistant against vancomycin (Fig. 2A). Uni- and multivariable regression analyses confirm that *E. faecium* isolates from the Northeast and Northwest regions are less likely to be tested vancomycin resistant than isolates from the Southwest (Table 2). Importantly, temporal dynamics of VREF proportions differ between the analysed regions (Fig. 2B). While in the Southwest and Southeast a pronounced increase of VREF proportions was observed between 2014 and 2017, the northern regions do not show a rise of VREF during that same period. In the Southwest and Southeast, VREF proportions increased from 10.8% (95% CI 6.9–16.5%) and 3.8% (95% CI 3.0–11.5%) in 2014 to 36.7% (95% CI 32.9–40.8%) and 36.8% (95% CI 29.2–44.7%) in 2017, respectively. This finding is supported by a multivariable analysis controlling for interaction between year and region (Additional file 3: Table S2). It is important to note that southern regions feature considerably higher VREF proportions than northern regions only from 2016 onwards.

The absolute number of VREF isolates in the ARS database continuously increased from 631 to 1634 between 2012 and 2017, suggesting that numbers of infections with vancomycin-resistant *E. faecium* in German hospitals have been annually increasing. This trend is supported by our analysis of publicly available data from the hospital payment system based on fee-for-case on DRGs [20] that show a four-fold increase of diagnoses of glycopeptide-resistant *E. faecium* infections or colonisations in German hospitals between 2013 and 2017 (Table 3).

**Table 3:** Analyses of diagnoses of glycopeptide-resitant *E. faecium* in German hospitals

*Year*	*Number of diagnoses*
2013	7074
2014	8488
2015	11,697
2016	19,747
2017	28,907

The number of diagnoses of infections or colonisations with glycopeptide-resistant *E. faecium* between 2013 and 2017 were analysed using publicly available data from the hospital payment system based on fee-for-case on diagnosis related groups. The dataset contains diagnosis data of ~ 1500 out of a total of 1924 existing (2017) German hospitals. The diagnoses code U80.30! according ICD-10-GM was used to identify cases of *E. faecium* with resistance to glycopeptide antibiotics

### Age and gender

**Fig. 3:**
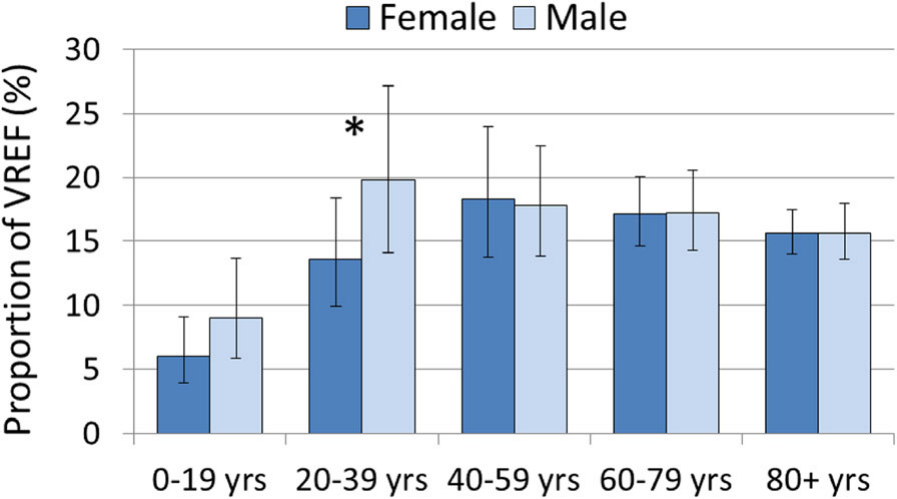
Vancomycin-resistant *E. faecium* stratified into age and gender. Vancomycin-resistant *E. faecium* (VREF) as a proportion (%) of all *E. faecium* isolates with corresponding 95% confidence intervals from male and female patients stratified into age categories. Proportions of VREF between female and male were compared using the Pearson χ^2^ test with the Rao-Scott second-order correction in different age groups. The resulting p-values were adjusted for multiple testing using a Bonferroni correction. P-values ≤0.05 are indicated with an “*”. Adjusted p-Values (female vs. male patients): 0–19 yrs.: *p* = 0.768, 20–39 yrs.: *p* = 0.013, 40–59 yrs.: p = 1, 60–79 yrs.: *p* = 1, 80+ yrs.: p = 1

In order to study the influence of the patient age on vancomycin resistance patterns, VREF proportions were analysed for different age categories. The results displayed in Fig. 3 show that *E. faecium* isolates from children and adolescents (0–19 years) exhibit markedly lower vancomycin resistance proportions (7.6% [95% CI 5.4–10.6%]) than young adults (20–39 years) (15.4% [95% CI 11.1–21.0%]) and older age categories. Multivariable analyses reveal that *E. faecium* samples from patient age categories of 0–19 years and 20–39 years are less likely to be vancomycin-resistant than samples from patients with an age between 40 and 59 years (Table 2). Interestingly, the likelihood of vancomycin resistance tends to decrease in age groups above 60 years, although the differences are not statistical significant. Further analyses of all age categories combined do not indicate that female and male patients differ in VREF proportions (16.5 [95% CI 14.2–19.1%] vs. 16.9 [95% CI 14.1–20.1%]), respectively. However, male young adults (20–39 years) have higher proportions of vancomycin resistance than their female counterparts (19.8 vs. 13.6%, adjusted *p*-value: 0.013), while no differences between both genders were observed in older age categories (Fig. 3).

### Clinical specimen

Since the frequency of drug-resistance pathogens can differ between infection sites, VREF proportions were analysed in different clinical specimens, including blood cultures, urine samples, wound material and swabs. No major differences in vancomycin resistance proportions were found between the analysed sampling sites (blood: 14.9% [95% CI 11.3–19.6%], urine: 17.2% [95% CI 14.6–20.2%], wound: 16.5% [95% CI 13.6–19.9%], swabs: 16.1% [95% CI 13.5–19.0%], other: 15.5% [95% CI 12.2–19.5%]). Therefore, no associations between clinical specimen and the likelihood of VREF resistance were found in uni- and multivariable regression analyses (Table 2).

### Hospital care type

**Fig. 4:**
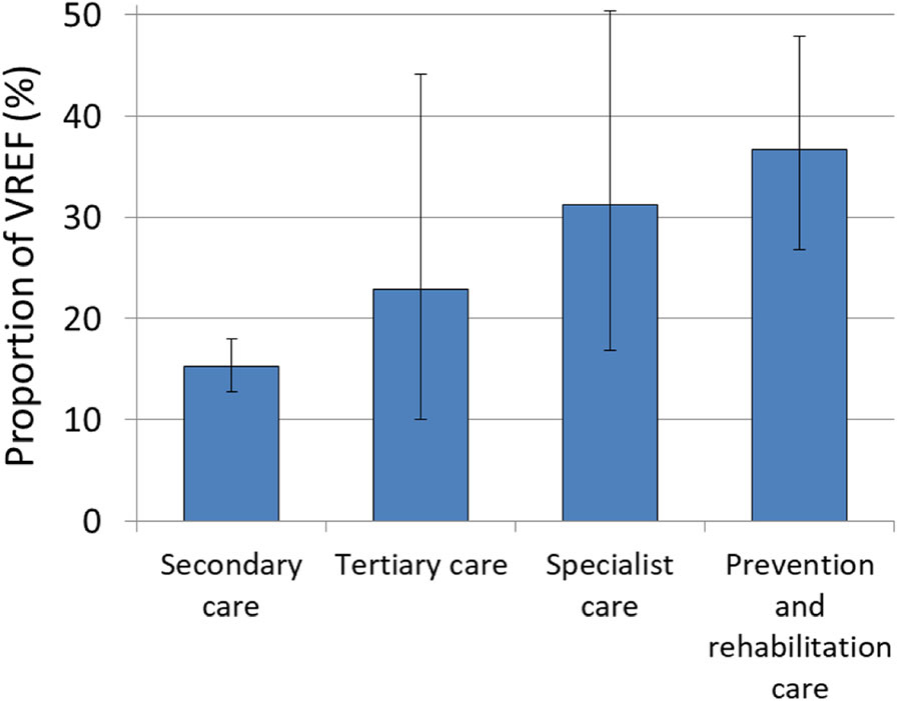
Vancomycin-resistant *E. faecium* stratified into hospital care type. Vancomycin-resistant *E. faecium* as a proportion (%) of all *E. faecium* isolates with corresponding 95% confidence intervals by hospital care type

To study vancomycin resistance patterns in different hospital care types, VREF proportions were analysed for secondary care, tertiary care and specialist care hospitals as well as prevention and rehabilitation care centres. *E. faecium* isolates from secondary care hospitals exhibited lower proportions of vancomycin resistance (15.2% [95% CI 12.8–18.0%]) than isolates from tertiary care hospitals (22.8% [95% CI 10.0–44.1%]) and specialist care hospitals (31.2% [95% CI 16.9–50.4%]) (Fig. 4). Univariable analyses show that *E. faecium* samples from tertiary hospital care and specialist hospital care are more likely to exhibit vancomycin resistance than isolates from secondary care (Table 2). However, in multivariable analyses, no statistical evidence was found that VREF proportions differ between secondary and tertiary care hospitals (*p* = 0.191). Interestingly, remarkably high proportions of VREF were observed in isolates from patients treated in prevention and rehabilitation care centres, where more than one third of all *E. faecium* isolates are found to be resistant to vancomycin (36.7% [CI 95% 26.8–47.9%]. In line with that, the multivariable regression analysis identified prevention and rehabilitation care centres as an independent risk factor of increased likelihood of VREF resistance in relation to secondary care hospitals.

## Discussion

By analysing data from the German *Antibiotic Resistance Surveillance* system the present study shows that from 2014 onwards the proportions of clinical *E. faecium* isolates exhibiting resistance to vancomycin increased from 11 to 26% in 2017. Regional analyses reveal that, in particular, southern regions of Germany have been affected by a pronounced rise of VREF proportions, whereas northern regions do not feature substantial increases of VREF. Middle aged adults (40–59 years) exhibit markedly higher VREF proportions than children and adolescents (0–20 years) and young adults (20–39 years). While VREF proportions do not differ between female and male patients in the whole dataset, subgroup analyses show that *E. faecium* isolates from young adult men have higher vancomycin resistance proportions than their female counterparts.

Rising proportions of vancomycin-resistant *E. faecium* have also been observed in many other European countries between 2014 and 2017 as reported by EARS-Net, including neighbouring countries to Germanys, such as Denmark, Belgium, Poland and Czech Republic [12]. Only two countries, Ireland and Portugal, in the EU and European Economic Area (EU/EEA) show a decreasing trend of VREF proportions between 2014 and 2017 (Portugal 20.1 to 7.2%, Ireland 45.1 to 38.2%). In EARS-Net AMR data are exclusively collected from invasive isolates. In line with the findings from EARS-Net our analyses of VREF proportions in blood isolates also show increasing trends between 2014 and 2017 in German hospitals.

Since infections with VREF are associated with worse clinical outcomes compared to infections with vancomycin-sensitive strains [21–23], rising vancomycin resistance is of great clinical concern in the management of patients with nosocomial *E. faecium* infections. As a matter of fact, a recent population-level study using data from EARS-Net showed that there were about 16,000 nosocomial infections with *vancomycin-resistant enterococci*, which were associated with 1065 attributable deaths in the EU/EEA in the year 2015, nearly twice as many as 2007 [24]. Current German data show increasing trends of nosocomial infections with vancomycin-resistant Enterococci in German hospitals [13, 25]. These findings are strongly supported by our analyses of publicly available data from German hospitals, which show a four-fold increase of diagnoses of infections or colonisations with glycopeptide-resistant *E. faecium* between 2013 and 2017 underlining the growing significance of vancomycin-resistant *E. faecium* in Germany. It is important to note that rising numbers of diagnoses of infections or colonisations with glycopeptide-resistant *E. faecium* may be partly explained by increased screening efforts in German hospitals, although no representative information about the development of VRE(F) screening habits are available. However, in Germany, the *Commission for Hospital Hygiene and Infection Prevention* only recommends a VRE(F) screening for risk populations (e.g. patients with severe comorbidities and haematological diseases) rather than a general screening for all hospitalized patients [26]. Rising numbers of infections with vancomycin-resistant enterococci and/or *E. faecium* have been also reported for other countries around the world, including Switzerland [27], Australia [28] and Canada [29, 30]. Interestingly, our data show that the ratio of clinical *E. faecium* and *E. faecalis* isolates recorded in ARS is higher in West and South Germany suggesting a more prominent role of *E. faecium* in enterococcal infections in these regions.

Analyses of resistance trends between different geographical regions in Germany between 2012 and 2017 reveal that VREF proportions significantly vary within Germany exhibiting a pronounced north-south disparity. While VREF proportions remained stable in northern regions, a marked increase of VREF proportions was observed in hospitals in the Southwest. From 2016 onwards VREF proportions in the South were significantly higher than in the North. VREF strain characterization based on whole genome sequencing performed at the *National Reference Centre for Enterococci* for all bloodstream isolates between 2015 and 2018 (*n* = 448) revealed prevalence of certain strain types associated to specific regions. Whereas ST117/CT71 was mainly spread throughout Germany and found in at least nine Federal States, isolates of ST117/CT469, ST80/CT1065 and ST80/CT1066 were mainly prevalent in Southwestern Germany ([7] and Jennifer K. Bender und Guido Werner, unpublished data). Therefore, the increase in VREF rates in certain regions in Germany might be associated with a preferred prevalence of certain strain types.

Increasing proportions of *enterococci* infections with vancomycin-resistant strains in Germany are also observed in data from the national *Nosocomial infection surveillance system* (KISS). In contrast to our findings, KISS identified a belt of states with higher proportions of vancomycin-resistant *enterococci* infections in the centre of Germany spanning from west to east [13]. The different results to our surveillance system might be explained by different methodological approaches used in the KISS study, such as only inclusion of bloodstream and urinary tract infections from ICUs and wound infections from surgical departments. The reasons for the regional differences observed in our study are largely unknown. However, a large representative population-based study analysing German antibiotic prescription data reported higher outpatient antibiotic prescription of fluoroquinolones in southwestern regions of Germany [31]. The extensive use of fluoroquinolones has been shown to be associated with the emergence of vancomycin-resistant enterococci in the hospital setting [32]. This finding underlines the importance of the implementing of interventions that improve outpatient antibiotic prescribing [33]. It is important to note that the analyses of regional resistance patterns are based on the location of the hospital rather than the residence of the patient. Nevertheless, hospital density in Germany is relatively high and it has been reported that the majority of patients are treated in hospitals fewer than 60 km from the patients places of residence [34]. This suggests that the described regional VREF proportions are a true reflection of the acquisition of VREF in the respective regions, irrespective of whether they were acquired in the hospital or in the community.

Very little is known about factors associated with increased vancomycin resistance in clinical *E. faecium* isolates in Germany. This study did not find any differences in VREF proportions between female and male patients. This finding is also reported in other studies from different regions in the world [35–38]. In contrast, a study analysing data from three New York hospitals found that isolates from female patients have a higher likelihood of being vancomycin-resistant than samples from men [39]. However, that particular study analysed infections with *Enterococcus faecalis* or *Enterococcus faecium.* Interestingly, we observed that young male adults (20–39 years) exhibit markedly higher proportions of VREF than young female adults (20% vs. 14%), a finding that has not been described for *E. faecium* yet.

Since it has been known that different age groups exhibit different microbial susceptibility proportions, VREF resistance patterns were analyzed for different age categories. Patients older than 40 years exhibit higher VREF proportions than children and adolescents (> 15% vs. 7%). Similar age trends have been reported for other bacterial pathogens, including *Staphylococcus aureus*, *Escherichia coli*, *Streptococcus pneumoniae*, *Pseudomonas aeruginosa, Helicobacter pylori* and *Klebsiella pneumonia* [16, 40–42]. A possible explanation is that older patients are more likely to be colonised with drug-resistant pathogens due to more frequent exposure to antibiotics throughout their lives, thereby promoting the selection of drug-resistant bacteria as described for enterococci [43]. In addition, in comparison to younger patients elderly patients are likely to have more comorbidities and are more likely to reside in nursing homes or other healthcare facilities, both factors that have been shown to be associated with increased antibiotic resistance [44].

Since nosocomial bloodstream infections are of particular public health relevance and are often associated with worse outcomes than other infection types [45–48], VREF proportions were analysed in clinical blood samples and other specimen. Blood samples do not show higher VREF proportions compared with urine samples, wound material and swabs. Interestingly, it has been shown that vancomycin resistance does not further increase the risk of in-hospital mortality and infection-attributed hospital stay in bloodstream infections with *E. faecium* but is associated with increased overall hospital costs [49].

This study indicates that VREF proportions are higher in specialist care hospitals and prevention and rehabilitation care centres, a finding that is possibly explained by the larger number of patients with comorbidities and other factors (e. g. age) that are associated with acquiring resistant bacteria. Specialist care hospitals and prevention and rehabilitation care centres have also been identified as risk factors for antimicrobial resistance in *Klebsiella pneumoniae* in Germany [16].

### Strengths and limitations

This study used data from the ARS database which is the largest and most comprehensive surveillance system for antimicrobial resistance in Germany [14, 50]. As of 2017, ARS comprised of data from more than 600 participating hospitals across all regions in Germany allowing for detailed analyses of epidemiological trends. To our knowledge, with more than 35.000 clinical isolates of *E. faecium* collected from more than 33,000 patients our study represents the most comprehensive analysis of recent trends of VREF in German hospitals. However, it is important to consider the limitations of this study. First, participation in ARS is voluntary, and thus, participating laboratories and hospitals are not equally distributed resulting in a clustering in certain regions. In particular, northern regions are under-represented in the sample set, while the Western region is overrepresented. Therefore, statistical analyses were used that accounted for clustering effects. Second, since information on underlying diagnoses is not collected in ARS, it is not possible to differentiate between colonisation and infection. To address this issue, isolates were excluded if they were likely collected for screening purposes. Third, although the analyses were restricted to hospitals that continuously participated in ARS between 2012 and 2017, it cannot ruled out that changes in hospital structures and case mix might have biased the longitudinal observations results. To account for these limitations the key finding of increasing VREF proportion and different regional patterns were confirmed by sensitivity analyses and regression analyses assessing the interaction between region and year which underlines the robustness of the results presented in this study.

## Conclusion

Proportions of vancomycin resistance in clinical *E. faecium* isolates from German hospitals are increasing underlining the growing significance of *E. faecium* infections for public health. VREF proportions differ considerable among German regions with a particular focus of high vancomycin resistance in Southwest and Southeast Germany. Continued surveillance and implementation of effective infection prevention and control measures accounting for local resistance differences are needed to reduce the spread of vancomycin-resistant *E. faecium* in German hospitals.

## Additional files

Additional file 1: **Table S1.** Ratio between the total numbers of *E. faecium* and *E. faecalis* isolates in the ARS database from continuously and non-continuously participating hospitals. (DOCX 16 kb)

Additional file 2: **Figure S1.** Sensitivity analyses of time trend of vancomycin-resistant *E. faecium. (DOCX 91 kb)*

Additional file 3: **Table S2.** Multivariable regression analysis assessing the interaction between region and year of sampling. (DOCX 18 kb)

## Data Availability

Aggregated ARS data are available online (https://ars.rki.de). All raw data can be provided on reasonable request.

## Abbreviations

AMR: Antimicrobial Resistance
ARS: Antimicrobial Resistance Surveillance
CI: Confidence intervals
CLSI: Clinical & Laboratory Standards Institute
EARS-Net: European Antimicrobial Resistance Surveillance Network
EUCAST: European Committee on Antimicrobial Susceptibility Testing
ICD-10-GM: International Statistical Classification of Diseases and Related Health Problems Version 10 - German Modification
IQR: interquartile range
KISS: *Nosocomial Infection Surveillance System*
OR: Odds ratio
VREF: Vancomycin-resistant *Enterococcus faecium*

## References

[1] Fisher K, Phillips C (2009). The ecology, epidemiology and virulence of Enterococcus. Microbiology.

[2] Arias CA, Murray BE (2012). The rise of the Enterococcus: beyond vancomycin resistance. Nat Rev Microbiol.

[3] Weiner LM (2016). Antimicrobial-resistant pathogens associated with healthcare-associated infections: summary of data reported to the National Healthcare Safety Network at the Centers for Disease Control and Prevention, 2011-2014. Infect Control Hosp Epidemiol.

[4] Behnke M (2013). Nosocomial infection and antibiotic use: a second national prevalence study in Germany. Dtsch Arztebl Int.

[5] Lee T (2019). Antimicrobial-resistant CC17 Enterococcus faecium: the past, the present and the future. J Glob Antimicrob Resist.

[6] Lebreton F, et al. Emergence of epidemic multidrug-resistant Enterococcus faecium from animal and commensal strains. MBio. 2013;4(4). 10.1128/mBio.00534-13 PMC3747589 23963180

[7] Liese J, et al. *Expansion of Vancomycin-Resistant Enterococcus faecium in an Academic Tertiary Hospital in Southwest Germany: a Large-Scale Whole-Genome-Based Outbreak Investigation*. Antimicrob Agents Chemother. 2019;**63**(5). 10.1128/AAC.01978-18 PMC6496047 30782988

[8] O'Driscoll T, Crank CW (2015). Vancomycin-resistant enterococcal infections: epidemiology, clinical manifestations, and optimal management. Infect Drug Resist.

[9] Hollenbeck BL, Rice LB (2012). Intrinsic and acquired resistance mechanisms in enterococcus. Virulence.

[10] Werner G, et al. *Emergence and spread of vancomycin resistance among enterococci in Europe*. Euro Surveill. 2008;**13**(47). 19021959

[11] Tacconelli E (2018). Discovery, research, and development of new antibiotics: the WHO priority list of antibiotic-resistant bacteria and tuberculosis. Lancet Infect Dis.

[12] Control, E.C.f.D.P.a., *Surveillance of antimicrobial resistance in Europe – Annual report of the European Antimicrobial Resistance Surveillance Network (EARS-Net) 2017*, in *Stockholm: ECDC*. 2018.

[13] Remschmidt C (2018). Continuous increase of vancomycin resistance in enterococci causing nosocomial infections in Germany - 10 years of surveillance. Antimicrob Resist Infect Control.

[14] Noll I (2012). Antimicrobial resistance in Germany. Four years of antimicrobial resistance surveillance (ARS). Bundesgesundheitsblatt Gesundheitsforschung Gesundheitsschutz.

[15] Schweickert B (2012). MRSA-surveillance in Germany: data from the antibiotic resistance surveillance system (ARS) and the mandatory surveillance of MRSA in blood. Eur J Clin Microbiol Infect Dis.

[16] Koppe U (2018). Carbapenem non-susceptibility of Klebsiella pneumoniae isolates in hospitals from 2011 to 2016, data from the German antimicrobial resistance surveillance (ARS). Antimicrob Resist Infect Control.

[17] Huang SS (2007). Improving the assessment of vancomycin-resistant enterococci by routine screening. J Infect Dis.

[18] Team, R.C., *R: A language and environment for statistical computing. R Foundation for Statistical Computing.* Available online at https://www.R-project.org/. 2018.

[19] Rao, J.N.K. and A. Scott, *On Chi-squared Tests For Multiway Contigency Tables with Proportions Estimated From Survey Data*. Vol. 12. 1984.

[20] *Datenlieferung gem. § 21 KHEntgG*. 2019 [cited 2019 March 15th 2019]; Available from: https://www.g-drg.de/Datenlieferung_gem._21_KHEntgG.

[21] Prematunge C (2016). VRE and VSE bacteremia outcomes in the era of effective VRE therapy: a systematic review and meta-analysis. Infect Control Hosp Epidemiol.

[22] Linden PK (1996). Differences in outcomes for patients with bacteremia due to vancomycin-resistant Enterococcus faecium or vancomycin-susceptible E. faecium. Clin Infect Dis.

[23] Garbutt JM (2000). Association between resistance to vancomycin and death in cases of Enterococcus faecium bacteremia. Clin Infect Dis.

[24] Cassini A (2019). Attributable deaths and disability-adjusted life-years caused by infections with antibiotic-resistant bacteria in the EU and the European economic area in 2015: a population-level modelling analysis. Lancet Infect Dis.

[25] Behnke M (2017). The prevalence of nosocomial infection and antibiotic use in German hospitals. Dtsch Arztebl Int.

[26] Infektionsprävention, K.f.K.u., *Hygienemaßnahmen zur Prävention der Infektion durch Enterokokken mit speziellen Antibiotikaresistenzen.* Bundesgesundheitsblatt - Gesundheitsforschung - Gesundheitsschutz, 2018. **61**(10): p. 1310–1361. 10.1007/s00103-018-2811-2 30229318

[27] Buetti N (2019). Emergence of vancomycin-resistant enterococci in Switzerland: a nation-wide survey. Antimicrobial Resistance & Infection Control.

[28] Leong KWC (2018). Emergence of vancomycin-resistant Enterococcus faecium at an Australian hospital: a whole genome sequencing analysis. Sci Rep.

[29] Canada, P.H.A.o., *Canadian Antimicrobial Resistance - 2017 Report*, in *CANADIAN ANTIMICROBIAL RESISTANCE SURVEILLANCE SYSTEM*. 2018, Public Health Agency of Canada Ottawa, ON K1A 0K9.

[30] Canada, P.H.A.o., *Canadian Antimicrobial Resistance Surveillance System - Update* 2018, in *Canadian Antimicrobial Resistance Surveillance System*. 2018, Public Health Agency of Canada: Ottawa, Canada.

[31] Batzing-Feigenbaum J (2016). Outpatient Antibiotic Prescription. Dtsch Arztebl Int.

[32] Forstner C (2015). *Non-linear significant relationship between use of glycopeptides and isolation of vancomycin-resistant Enterococcus species in a university hospital setting*. Antimicrob Resist Infect Control.

[33] Dyar OJ (2016). How can we improve antibiotic prescribing in primary care?. Expert Rev Anti-Infect Ther.

[34] Klauber J, et al. Stuttgart, Germany. Krankenhaus-Report. 2015:2015.

[35] Moemen D, Tawfeek D, Badawy W (2015). Healthcare-associated vancomycin resistant Enterococcus faecium infections in the Mansoura University hospitals intensive care units*,* Egypt. Braz J Microbiol.

[36] Sabouni F (2016). High frequency of vancomycin resistant Enterococcus faecalis in children: an alarming concern. J Prev Med Hyg.

[37] Tripathi A (2016). Prevalence, outcome and risk factor associated with vancomycin-resistant Enterococcus faecalis and Enterococcus faecium at a tertiary Care Hospital in Northern India. Indian J Med Microbiol.

[38] Lucas GM (1998). Vancomycin-resistant and vancomycin-susceptible enterococcal bacteremia: comparison of clinical features and outcomes. Clin Infect Dis.

[39] Monteserin N, Larson E (2016). Temporal trends and risk factors for healthcare-associated vancomycin-resistant enterococci in adults. J Hosp Infect.

[40] Adam HJ (2013). Comparison of pathogens and their antimicrobial resistance patterns in paediatric, adult and elderly patients in Canadian hospitals. J Antimicrob Chemother.

[41] Ji Z (2016). The Association of age and Antibiotic Resistance of helicobacter pylori: a study in Jiaxing City, Zhejiang Province, China. Medicine (Baltimore).

[42] Garcia A, Delorme T, Nasr P (2017). Patient age as a factor of antibiotic resistance in methicillin-resistant Staphylococcus aureus. J Med Microbiol.

[43] Karki S (2012). Prevalence and risk factors for VRE colonisation in a tertiary hospital in Melbourne, Australia: a cross sectional study. Antimicrob Resist Infect Control.

[44] Laudisio A (2017). The burden of comorbidity is associated with antibiotic resistance among institutionalized elderly with urinary infection: a retrospective cohort study in a single Italian nursing home between 2009 and 2014. Microb Drug Resist.

[45] Koch AM (2015). Mortality related to hospital-associated infections in a tertiary hospital; repeated cross-sectional studies between 2004-2011. Antimicrob Resist Infect Control.

[46] Goto M, Al-Hasan MN (2013). Overall burden of bloodstream infection and nosocomial bloodstream infection in North America and Europe. Clin Microbiol Infect.

[47] Klevens RM (2007). Estimating health care-associated infections and deaths in U.S. hospitals, 2002. Public Health Rep.

[48] Alon D (2013). Predictors and outcomes of infection-related hospital admissions of heart failure patients. PLoS One.

[49] Kramer TS (2018). The importance of adjusting for enterococcus species when assessing the burden of vancomycin resistance: a cohort study including over 1000 cases of enterococcal bloodstream infections. Antimicrob Resist Infect Control.

[50] Noll I (2018). Antibiotic consumption and antimicrobial resistance in human and veterinary medicine : an overview of established national surveillance systems in Germany. Bundesgesundheitsblatt Gesundheitsforschung Gesundheitsschutz.

