# Supplementary material for: The rise in vancomycin-resistant Enterococcus faecium in Germany: data from the German Antimicrobial Resistance Surveillance (ARS)
Source: Antimicrob Resist Infect Control. 2019 Aug 28;8:147. doi: 10.1186/s13756-019-0594-3 (PMC6712849; doi:10.1186/s13756-019-0594-3)
Supplement: Supplementary file 1 — Table S1. Ratio between the total numbers of E. faecium and E. faecalis isolates in the ARS database from continuously and non-continuously participating hospitals. (DOCX 16 kb) [file 13756_2019_594_MOESM1_ESM.docx]

***Additional file 1***

**Supplementary table 1. Ratio between the total numbers of *E. faecium* and *E. faecalis* isolates in the ARS database from continuously and non-continuously participating hospitals**

| **Ratio *E. faecium* / *E. faecalis* (No. Isolates in ARS *E. faecium* / *E. faecalis*)** | | |
| --- | --- | --- |
|  | **2012** | |
| Southwest | 0.40 | (3,344 / 8,369) |
| Southeast | 0.32 | (368 / 1,168) |
| West | 0.40 | (5,274 / 13,114) |
| Northwest | 0.38 | (1,247 / 3,320) |
| Northeast | 0.18 | (85 / 462) |
| **Germany (total)** | **0.39** | **10,318 / 26,433)** |
|  |  |  |
|  | **2013** | |
| Southwest | 0.41 | (3.986 / 9,724) |
| Southeast | 0.30 | (221 / 746) |
| West | 0.39 | (6,597 / 16,933) |
| Northwest | 0.32 | (730 / 2,312) |
| Northeast | 0.21 | (891 / 4,318) |
| **Germany (total)** | **0.37** | **(12,425 / 34,033)** |
|  |  |  |
|  | **2014** | |
| Southwest | 0.45 | (2,644 / 5,848) |
| Southeast | 0.25 | (816 / 3,307) |
| West | 0.38 | (4,771 / 12,519) |
| Northwest | 0.31 | (801 / 2,607) |
| Northeast | 0.21 | (882 / 4,199) |
| **Germany (total)** | **0.35** | **(9,914 / 28,480)** |
|  |  |  |
|  | **2015** | |
| Southwest | 0.52 | (2,406 / 4,626) |
| Southeast | 0.26 | (2,736 / 10,391) |
| West | 0.40 | (7,288 / 47,123) |
| Northwest | 0.25 | (1,817 / 6,825) |
| Northeast | 0.24 | (1,626 / 6,719) |
| **Germany (total)** | **0.34** | **(15,873 / 47,123)** |
|  |  |  |
|  | **2016** | |
| Southwest | 0.45 | (3,295 / 7,267) |
| Southeast | 0.27 | (3,547 / 13,318) |
| West | 0.45 | (7,291 / 16,120) |
| Northwest | 0.26 | (1,796 / 6,825) |
| Northeast | 0.26 | (1,592 / 6,035) |
| **Germany (total)** | **0.35** | **(17,521 / 49,565)** |
|  |  |  |
|  | **2017** | |
| Southwest | 0.34 | (4,297 / 12,499) |
| Southeast | 0.29 | (5,029 / 17,597) |
| West | 0.53 | (7,407 / 13,919) |
| Northwest | 0.30 | (3,259 / 10,816) |
| Northeast | 0.28 | (3,381 / 12,277) |
| **Germany (total)** | **0.35** | **(23,373 / 67,108)** |
