# Supplementary material for: The rise in vancomycin-resistant Enterococcus faecium in Germany: data from the German Antimicrobial Resistance Surveillance (ARS)
Source: Antimicrob Resist Infect Control. 2019 Aug 28;8:147. doi: 10.1186/s13756-019-0594-3 (PMC6712849; doi:10.1186/s13756-019-0594-3)
Supplement: Supplementary file 2 — Figure S1. Sensitivity analyses of time trend of vancomycin-resistant E. faecium. (DOCX 91 kb) [file 13756_2019_594_MOESM2_ESM.docx]

***Additional file 2***


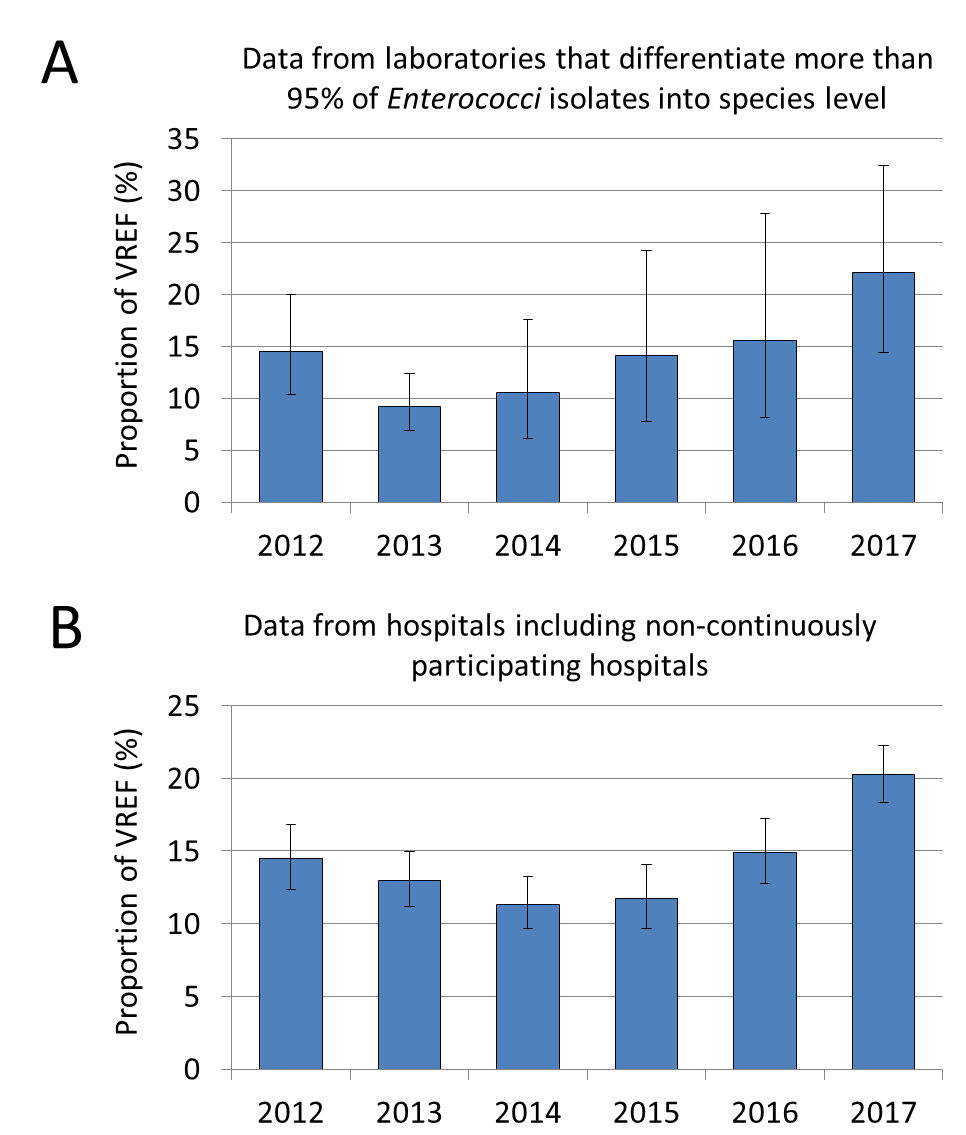


**Supplementary figure 1. Sensitivity analyses of time trend of vancomycin-resistant *E.  faecium***

Time trend of vancomycin-resistant E. faecium as a proportion (%) of all E. faecium isolates with corresponding 95% confidence intervals. VREF proportions were analysed among isolates from laboratories that routinely differentiate more than 95% of enterococci isolates into species levels (n = 8,492) (A) and among isolates from all hospitals including hospitals that did not continuously participated in ARS between 2012 and 2017 (n = 89,450) (B).
