# Supplementary material for: The rise in vancomycin-resistant Enterococcus faecium in Germany: data from the German Antimicrobial Resistance Surveillance (ARS)
Source: Antimicrob Resist Infect Control. 2019 Aug 28;8:147. doi: 10.1186/s13756-019-0594-3 (PMC6712849; doi:10.1186/s13756-019-0594-3)
Supplement: Supplementary file 3 — Table S2. Multivariable regression analysis assessing the interaction between region and year of sampling. (DOCX 18 kb) [file 13756_2019_594_MOESM3_ESM.docx]

***Additional file 3***

**Supplementary table 2. Multivariable regression analysis assessing the interaction between region and year of sampling.**

|  |  | multivariable analysis | | |
| --- | --- | --- | --- | --- |
|  |  | OR | (95% CI) | p-value |
| *Region* | |  |  |  |
|  | Southwest | 1 | - | - |
|  | Southeast | 0.35 | (0.18-0.65) | 0.001 |
|  | West | 2.34 | (1.40-3.90) | 0.002 |
|  | Northwest | 1.91 | (1.11-3.30) | 0.021 |
|  | Northeast | 1.38 | (0.81-2.37) | 0.242 |
| *Year (per unit from 2012)* | | 1.42 | (1.31-1.54) | <0.001 |
| *Region x Year (per unit from 2012)* | | | | |
|  | Southwest | 1 | - | - |
|  | Southeast | 1.21 | (1.03-1.43) | 0.020 |
|  | West | 0.69 | (0.63-0.76) | <0.001 |
|  | Northwest | 0.62 | (0.54-0.71) | <0.001 |
|  | Northeast | 0.71 | (0.59-0.85) | <0.001 |
| *Gender* | |  |  |  |
|  | Female | 1.00 | - | - |
|  | Male | 1.03 | (0.94-1.11) | 0.517 |
| *Age* | |  |  |  |
|  | 0-19 years | 0.32 | (0.22-0.47) | <0.001 |
|  | 20-39 years | 0.85 | (0.71-1.01) | 0.074 |
|  | 40-59 years | 1 | - | - |
|  | 60-79 years | 0.94 | (0.83-1.05) | 0.263 |
|  | 80+ years | 0.83 | (0.69-1.00) | 0.048 |
| *Specimen (sampling site)* | |  |  |  |
|  | Blood | 1 | - | - |
|  | Urine | 1.19 | (0.92-1.52) | 0.187 |
|  | Swab | 0.92 | (0.74-1.16) | 0.496 |
|  | Wound | 1.04 | (0.88-1.22) | 0.661 |
|  | Other | 0.99 | (0.84-1.17) | 0.885 |
| *Hospital care type* | |  |  |  |
|  | Secondary care | 1 | - | - |
|  | Tertiary care | 1.38 | (0.90-2.11) | 0.144 |
|  | Specialist care | 2.41 | (1.33-4.38) | 0.004 |
|  | Prevention and rehabilitation care | 2.43 | (1.91-3.07) | <0.001 |
|  |  |  |  |  |

Year was treated as a continuous predictor and the interaction between region and year was included. In order to calculate the influence of a given region and year on the odds ratio, the coefficient for region is multiplied with the coefficient for year (per unit) to the power (chosen year – 2012), and finally multiplied with the coefficient region x year to the power (chosen year – 2012), i.e.

*(region * year(per unit)*^(chosen year^ *^– 2012)^) * (region x year(per unit)* ^(chosen year^ *^– 2012)^).*

Example: In order to analyse the odds ratio of the Southeast in 2015 in relation to the Southwest in 2012 (with the same isolate characteristics) following calculation is performed: (0.35 * 1.42^(2015 – 2012)^) * (1.21 ^(2015 – 2012)^).
